# L-leucine increases the sensitivity of drug-resistant *Salmonella* to sarafloxacin by stimulating central carbon metabolism and increasing intracellular reactive oxygen species level

**DOI:** 10.3389/fmicb.2023.1186841

**Published:** 2023-05-12

**Authors:** Heng Yang, Yanhong Zhou, Qiong Luo, Chunyang Zhu, Binghu Fang

**Affiliations:** ^1^Guangdong Provincial Key Laboratory of Veterinary Pharmaceutics Development and Safety Evaluation, South China Agricultural University, Guangzhou, China; ^2^National Risk Assessment Laboratory for Antimicrobial Resistance of Animal Original Bacteria, South China Agricultural University, Guangzhou, China

**Keywords:** L-leucine, sarafloxacin, *salmonella*, metabolism, reactive oxygen species

## Abstract

**Introduction:**

The overuse of antibiotics has made public health and safety face a serious cisis. It is urgent to develop new clinical treatment methods to combat drug resistant bacteria to alleviate the health crisis. The efficiency of antibiotics is closely related to the metabolic state of bacteria. However, studies on fluoroquinolone resistant *Salmonella* are relatively rare.

**Methods:**

CICC21484 were passaged in medium with and without sarafloxacin and obtain sarafloxacin- susceptible *Salmonella Typhimurium* (SAR-S) and sarafloxacin resistant *Salmonella Typhimurium* (SAR-R), respectively. Non-targeted metabolomics was used to analyze the metabolic difference between SAR-S and SAR-R. Then we verified that exogenous L-leucine promoted the killing effect of sarafloxacin in vitro, and measured the intracellular ATP, NADH and reactive oxygen species levels of bacteria. Gene expression was determined using Real Time quantitative PCR.

**Results:**

We confirmed that exogenous L-leucine increased the killing effect of sarafloxacin on SAR-R and other clinically resistant *Salmonella* serotypes. Exogenous L-leucine stimulated the metabolic state of bacteria, especially the TCA cycle, which increased the working efficiency of the electron transfer chain and increased the intracellular NADH, ATP concentration, and reactive oxygen species level. Our results suggest that when the metabolism of drug-resistant bacteria is reprogrammed, the bactericidal effect of antibiotics improves.

**Discussion:**

This study further enhances research in the anti-drug resistance field at the metabolic level and provides theoretical support for solving the current problem of sarafloxacin drug resistance, a unique fluoroquinolone drug for animals and indicating the potential of L-leucine as a new antibiotic adjuvant.

## 1. Introduction

*Salmonella* is a zoonotic pathogen that can cross-spread among humans, animals, and the environment. It has important biological significance in gastrointestinal diseases (Ao et al., 2015; Gut et al., 2018). There are more than 900,000 cases of *Salmonella* infection worldwide each year, with 15,500 deaths, which has caused a massive burden on public health and safety (Majowicz et al., 2010; Feasey et al., 2012; Kirk et al., 2015). After infection, the main symptoms are vomiting, fever, and diarrhea. Infection in infants with low immunity and the elderly may further lead to more severe bacteremia and endanger life safety, and a few may even experience meningitis (Yang et al., 2012). Livestock and poultry infected with *Salmonella* will spread the pathogen to humans through food, environmental pollution, or other ways. *Salmonella* in animals can also reduce the rate of weight gain, resulting in reduced production performance and increased feed conversion rate, causing economic losses (Farzan and Friendship, 2010). At present, more than 2,500 *Salmonella* serotypes that have been identified (Hendriksen et al., 2011), of which *Salmonella Typhimurium* is one of the typical representatives and the primary serotype in the detection of non-human *Salmonella* (Galanis et al., 2006). In Thailand and Vietnam, the detection rate of *Salmonella Typhimurium* is 34 and 37.5%, respectively, the most prevalent serotype (Phu Huong Lan et al., 2016; Sinwat et al., 2016). In sub-Saharan Africa, Typhimurium accounts for two-thirds of all serotypes (Reddy et al., 2010). In China, the prevalence of *Salmonella Typhimurium* has also attracted people’s attention to pig breeding (Tian et al., 2021).

Sarafloxacin is the third generation of fluoroquinolones. The most common preparation is sarafloxacin hydrochloride, which can kill both Gram-negative and Gram-positive bacteria (Hooper, 1998; Oliphant and Green, 2002). After entering the cell, sarafloxacin can combine with DNA cyclooxygenase and topoisomerase IV to form a “drug-enzyme-DNA” ternary complex, thus preventing bacterial DNA replication and killing bacteria (Correia et al., 2017). In 1995, the U.S. Food and Drug Administration approved sarafloxacin as a special quinolone antibiotic for animals, which has a good curative effect on clinical respiratory tract infections, urinary system infections, skin tissue infections, and intra-abdominal infections (Kim and Hooper, 2014). Unfortunately, the overuse of antibiotics during bacterial infection in aquaculture, directly or indirectly, has led to the emergence and prevalence of drug-resistant *Salmonella*. The detection rate of fluoroquinolone-resistant *Salmonella* has also gradually increased (Fang, 2015). In 2019, drug-resistant *Salmonella* serotype Typhi was defined as a serious threat (CDC, 2019). The common resistance mechanism of fluoroquinolones includes: (i) the target mutation of DNA gyrase and topoisomerase IV, which leads to a decreased affinity between drugs and enzymes (Aldred et al., 2014); (ii) the modification of cell membrane protein and drug efflux pumps OqxAB, QepA, and AcrAB-TolC, which reduces intracellular drug concentrations (Redgrave et al., 2014); and (iii) plasmid-mediated acquired drug resistance (*qnrA*, *qnrB*, *qnrS*, *qnrC*, and *qnrD*; Aldred et al., 2014). However, with the development and application of metabolomics, researchers can directly observe the metabolic state changes of cells caused by various mechanisms (Schrimpe-Rutledge et al., 2016), and the analysis of drug resistance from a metabolic perspective has gradually become a research hotspot.

The effect of antibiotics on bacteria is closely related to metabolic status (Stokes et al., 2019; Lopatkin et al., 2021). The metabolomic results of cefoperazone/sulbactam-resistant *Pseudomonas aeruginosa* have shown the inhibition of the central carbon and riboflavin metabolism, while the related glucose metabolism and the electric transport chain also decreased (Chen et al., 2022). The decompressed central carbon and energy metabolism led to levofloxacin resistance in *Vibrio algenolyticus*, and the biosynthesis of fatty acids was also affected (Cheng et al., 2018). These studies have proved that biochemical metabolism plays a key role in the drug-resistant characteristics of bacteria. Consistently reprogramming metabolism by exogenous addition of certain substances can also reverse the drug-resistant characteristics of bacteria. Exogenous D-ribose activates glycolysis, the pentose phosphate pathway, and the TCA cycle, and increases NADH production and proton motive force (PMF) and, thus, increases drug uptake and promotes the killing effect of gentamicin against drug-resistant *Salmonella*. Exogenous citrulline similarly promotes the germicidal effect of apramycin (Yong et al., 2021; Zhou et al., 2022). The abundance of glutamine in the metabolomics results decreased. After supplementation, exogenous glutamine promotes the killing effect of β-lactams, aminoglycosides, quinolones and tetracyclines on pathogenic *E. coli* in the urinary tract through various metabolic pathways, and delay the development of ampicillin resistance (Zhao et al., 2021).

One of the common characteristics of bactericidal antibiotics is that reactive oxygen species (ROS) will be produced after targeting bacteria (Kohanski et al., 2007). ROS is an inevitable by-product of cellular aerobic respiration. These molecules are produced due to excessive activation of the electron transfer chain and leakage of electrons to the oxygen molecule to generate superoxide (O_2_^−^) ( $O_{2}+e^{-}=O_{2}^{-}$ ). Superoxide dismutases can catalyze superoxide generation to hydrogen peroxide and oxygen ( $2H^{+}+2O_{2}^{-}=O_{2}+H_{2}O_{2}$ ). Superoxide can destroy the iron–sulfur cluster in protein, thus producing unstable ferrous ions (Fe^2+^). One of the products of the reaction of Fe^2+^ and hydrogen peroxide is the lethal hydroxyl radical ( $Fe^{2+}\;H_{2}O_{2}=Fe^{3+}+OH^{-}+HO^{*}$ ). Hydroxyl radicals can destroy cell DNA, lipids, and proteins, resulting in death (Belenky et al., 2015). At the same time, it will also destroy the iron sulfide cluster protein, regenerate free iron ions, and generate more hydroxyl radicals through the Fenton reaction to accelerate bacterial death (Van Acker and Coenye, 2017). Some studies have confirmed that the overactivated electron transfer chain is related to central carbon metabolism, as the production of NADH mainly comes from the TCA cycle (Kohanski et al., 2007). Exogenous thymine can up regulate the bacterial metabolic state, activate the TCA cycle, accelerate respiration, promote the production of ATP and ROS, and promote the killing effect of ciprofloxacin on some Gram-negative bacteria (Liu et al., 2021). The addition of exogenous serine has been shown to promote the TCA cycle of *E. coli*, increase the production of NADH and the ratio of NAD^+^/NADH, activate the electron transfer chain, and increase the production of endogenous ROS, making the killing effect of ofloxacin or moxifloxacin on *E.coli* stronger (Duan et al., 2016). Alanine can also promote kanamycin killing of antibiotic resistant bacteria by promoting ROS production (Ye et al., 2018). Therefore, linking the metabolic state of bacteria to the production of ROS and the increase of intracellular ROS levels after the drug has targeted drug-resistant bacteria by stimulating metabolism has great research potential.

In this study, non-targeted metabolomics was used to analyze the metabolic characteristics of SAR-R and SAR-S. We found that carbon metabolism and other related pathways, such as the TCA cycle, decreased in SAR-R. The addition of exogenous L-leucine promoted the killing effect of sarafloxacin against multidrug-resistant *Salmonella*. The specific mechanism included activating the TCA cycle and the electron transfer chain, thus cooperating with antibiotics to produce more ROS, which accelerated the death of bacteria. Our results provide theoretical support for alleviating the clinical drug resistance of fluoroquinolones, especially the special animal drug sarafloxacin.

## 2. Materials and methods

### 2.1. Chemicals

Sarafloxacin hydrochloride was purchased from the China Institute of Veterinary Drug Control (Beijing, China). L-leucine and thiourea were purchased from Shanghai Macklin Biochemical Technology Co., Ltd. (Shanghai, China). Mueller Hinton (MH) broth, tryptic soy agar (TSA), Luria-Bertani (LB) broth, MacConkey agar, and MH agar were purchased from Guangdong Huankai Microbial Sci & Tech Co., Ltd. (Guangdong, China). M9 Minimal medium was purchased from Shanghai ELITE Biotech Co., Ltd. (Shanghai, China). Methanol and acetonitrile (high-performance liquid chromatography grade) were purchased from Thermo Fisher Scientific (Waltham, MA, United States).

### 2.2. Bacterial strains

Standard strains of *E. coli* (ATCC25922) were purchased from the American Type Culture Collection (Manassas, VA, United States). Standard strains of *Salmonella Typhimurium* (CICC21484) were purchased from the China Center of Industrial Culture Collection (Beijing, China). Clinically resistant *Salmonella* strains (Typhimurium B2, Derby A2, London E1) were donated by the Guangdong Dahuanong Animal Health Products Co., Ltd. (Guangdong, China). Cells were cultured in LB medium at 37°C shaking at 200 rpm unless specified otherwise.

### 2.3. Minimum inhibitory concentration and induction of bacterial antibiotic resistance

The MICs of sarafloxacin against different *Salmonella* strains were determined using the microdilution method (Wiegand et al., 2008). All MICs were tested in duplicate at least twice. The increasing concentration method was used to induce drug-resistant strains. *Salmonella* strain CICC21484 was inoculated into the LB medium, and sarafloxacin was added at half of the MIC concentration. The MIC of the bacteria was rechecked every 3 days. Then, a new half MIC concentration was added to the medium until the MIC reached the breakpoint, and we obtained SAR-R. Concurrently *Salmonella Typhimurium* was obtained without adding drugs but undergoing the same continuous reproductive process as SAR-S.

### 2.4. Non-targeted metabolomic determination and analysis

SAR-S and SAR-R were used for metabolomic determination. Two strains were cultured in LB medium until the exponential stage. The bacterial suspension (10 mL) was then centrifuged at 4°C and 12,000 g for 10 min, and the precipitate was washed twice with PBS. The two collected bacterial strains quickly quenched in liquid nitrogen to stop their metabolism. Then, precooled methanol/acetonitrile/water solution (2:2:1, v/v) was added, mixed by vortexing, and subjected to low-temperature ultrasound for 30 min. The sample rested at 20°C for 10 min, followed by centrifugation at 14000 g and 4°C for 20 min. The supernatant was collected for vacuum drying, and 100 μL acetonitrile aqueous solution (acetonitrile: water = 1:1, v/v) was added for mass spectrometry and to redissolve the pellet. The contents were centrifuged at 4°C for 15 min, and the supernatant was collected for further analysis. The samples were separated by an Agilent 1,290 Infinity LC ultrahigh performance liquid chromatography (UHPLC) HILIC column. Specifications were: column temperature 25°C; flow rate 0.5 mL/min; injection volume 2 μL; mobile phase composition A: water +25 mM ammonium acetate +25 mM ammonia water; B: acetonitrile. The gradient elution procedure was as follows: 0–0.5 min, 95% B; 0.5–7 min, B changed linearly from 95 to 65%; 7–8 min, B changed linearly from 65 to 40%; 8–9 min, B was maintained at 40%; 9–9.1 min, B changed linearly from 40 to 95%; 9.1–12 min, B was maintained at 95%. The sample was placed in the 4°C automatic sampler during the analysis process. In order to avoid the impact caused by the fluctuation of the instrument detection signal, continuous analysis of samples was performed in random order. Quality control (QC) samples were inserted into the sample queue to monitor and evaluate the stability of the system and the reliability of the experimental data. The Electron Spray Ionization source conditions after HILIC chromatographic separation were as follows: ion source gas 1 (gas 1): 60; ion source gas 2 (gas 2): 60; curtain gas (CUR): 30; source temperature: 600°C, ionSapary voltage floating ±5,500 V (positive and negative modes); TOF MS scan m/z range: 60–1,000 Da; product ion scan m/z range: 25–1,000 Da; TOF MS scan accumulation time: 0.20 s/spectra; and product ion scan accumulation time: 0.05 s/spectra. The secondary mass spectrometry was obtained by information-dependent acquisition (IDA) and high sensitivity mode with a clustering potential of ±60 V (positive and negative modes) and collision energy at 35 ± 15 eV. The IDA settings were as follows: exclude isotopes within 4 Da; and candidate ions to monitor per cycle: 10.

The XCMS software was used for peak alignment, retention time correction, and peak area extraction. For the data extracted with the software, we first identified the metabolite structure, pretreated the data, and then evaluated the quality of the experimental data and analysis. All metabolites identified (metabolites identified by combining positive and negative ions) were then classified and counted according to chemical taxonomy attribution information. The data were analyzed using principal component analysis (PCA), partial least squares discriminant analysis (PLS-DA), orthogonal partial least squares discriminant analysis (OPLS-DA), multivariate analysis, and the Student’s *t*-test to study the intra- and inter-group differences between samples and to identify the different metabolites. The variable importance for the projection (VIP) obtained from the OPLS-DA model was used to measure the influence intensity and interpretation ability of each metabolite’s expression pattern on the classification and discrimination of each sample group in order to mine the different metabolic molecules with biological significance. We used OPLS-DA VIP > 1 and *p* value <0.05 as the screening criteria for significantly different metabolites for subsequent pathway analysis. The MetaboAnalyst and Heatmapper online website (https://www.metaboanalyst.ca/MetaboAnalyst/ and http://www.heatmapper.ca/) online tools were used for hierarchical clustering of differential metabolites.

### 2.5. Antibiotic survival assay

To assess the effect of L-leucine on the bactericidal activity of sarafloxacin against SAR-R and the other three clinical strains, The cells were first cultured in LB to reach the exponential phase, centrifuged at 12,000 g for 5 min, and washed twice with sterile PBS buffer. Cells were then resuspended to M9 at an initial concentration of 1 × 10^6^ colony-forming units (CFU)/mL. Experimental strains were treated with sarafloxacin (1-fold MIC: 16 μg/mL for SAR-R, 4 μg/mL for Typhimurium B2, 8 μg/mL for Derby A2, and 2 μg/mL for London E1), and with or without L-leucine (20 mM or other concentrations) for 8 h in M9 Minimal medium. In the ROS quenching experiment, 60 mM thiourea was added to the medium and incubated for 2 h at 37°C. After incubation, 100 μL of the sample was used in a dilution series, and 20 μL of each dilution was spread onto TSA agar plates. CFUs were counted after overnight (16 h) incubation. Percentage survival was determined by the ratio of CFU obtained from the test and control samples.

### 2.6. NADH measurements

The NADH measurement assay was performed as described previously (Yong et al., 2021). NADH production was determined using an NAD^+^/NADH Assay Kit (BioAssay Systems, Hayward, CA, United States). In brief, after culturing bacteria and L-leucine in M9 Minimal medium for 4 h, 1 mL of culture was centrifuged for 5 min at 13,000 g. The supernatant was discarded, and the pellet was washed and precipitated with PBS 3 times. Approximately 100 μL of NADH extraction buffer was added to the washed bacterial pellet and incubated at 60°C for 5 min. Then, 20 μL of the assay buffer and 100 μL of NAD extraction buffer were added. After centrifugation, the concentration of NADH was measured with a multi-function e-microplate reader.

### 2.7. ATP measurements

The ATP concentration was determined using an ATP Assay Kit (Beyotime, China). In brief, after co-culturing bacteria (1 × 10^6^ CFU/mL) and L-leucine for 4 h, 1 mL of the bacterial solution was centrifuged at 4°C and 12,000 g for 5 min. The supernatant was discarded, and the bacterial pellet was washed twice and resuspended in PBS. Because ATP is relatively stable at low temperatures, the following operations were performed in an ice bath. Approximately 200 μL of the cracking solution was added to the bacterial suspension and incubated for 15 min, with shaking during the incubation period to crack fully. The sample was then centrifuged at 4°C and 12,000 g for 5 min, and the supernatant was collected for subsequent detection. A luminometer was used to detect the ATP concentration.

### 2.8. ROS measurements

The ROS Assay Kit was used to measure the intracellular ROS level (Beyotime, China). In brief, 1 mL of bacterial solution in the exponential phase cells was loaded with a 10 μL fluorescent probe DCFH-DA. Samples were incubated in the dark for 20 min with gentle shaking to ensure loading efficiency. After incubation, the samples were centrifuged at 4°C and 12,000 g for 5 min. The supernatant was discarded, and the pellet was washed twice with PBS to ensure the probe was cleared. The washed bacteria were resuspended in M9 Minimal medium, the initial concentration was adjusted to 1 × 10^6^ CFU/mL, and cultured in sarafloxacin with or without L-leucine. The ROS level was measured at 488 nm excitation wavelength and 525 nm emission wavelength at 2 h. The ROS relative level is expressed by the ratio of the experimental group sample to the control sample.

### 2.9. Real time quantitative PCR

After co-culturing the bacteria and L-leucine for 6 h, 5 mL of the bacterial solution was collected by centrifugation and precipitated for subsequent experiments. The RNAiso Plus Kit (Takara Japan) was used to extract the total RNA according to the manufacturer’s instructions. The Hifair® II 1st Strand cDNA Synthesis Kit (Yeasen, Shanghai, China) and 2000 ng total RNA were used for RT-qPCR according to the manufacturer’s instructions. The RT-qPCR reaction setup included 5 μL SYBR Green Master Mix (Yeasen, Shanghai, China), forward primer (2 μM), reverse primer (2 μM), and 2 μL cDNA added to RNase-free water to a total volume of 10 μL. The reaction tube was briefly centrifuged to ensure all the reaction liquid was collected at the bottom. The cycle parameters were as follows: initial denaturation at 95°C for 5 min; and 40 cycles of denaturation at 95°C for 10 s, and annealing and extension at 60°C for 30 s. The heating rate was 0.05°C/s for 60–95°C, in which a melting curve was obtained. The 16S rRNA gene was used as an internal reference. The experiment was performed with 3 biological replicates. The specific primers are listed in the Supplementary material (Supplementary Table 1).

## 3. Results

### 3.1. Induction and growth characteristics of drug-resistant *Salmonella*

After standard strains of *Salmonella Typhimurium* (CICC21484) were continuously sub-cultured *in vitro* with half the MIC concentration of sarafloxacin, we obtained drug-resistant strain SAR-R. The MIC measurement for strain SAR-R was 16 μg/mL, indicating that the resistant bacteria were successfully induced. The MIC test results of the three clinically isolated *Salmonella* strains with different serotypes of drug resistance are shown in Table 1.

**Table 1:** MIC value of different antimicrobials against *Salmonella* (μg/mL).

	SAR-S	SAR-R	Typhimurium B2	Derby A2	Londom E1
Sarafloxacin	0.03	16	4	8	2
Ciprofloxacin	0.015	4	2	1	1
Enrofloxacin	0.03	8	8	4	4
Danooxacin	0.03	4	4	4	2
Gentamicin	0.25	0.25	64	16	64
Apramycin	2	2	128	128	64
Tobramycin	0.25	0.25	64	32	32
Ampicillin	2	2	128	256	64
Tetracycline	1	1	64	32	64

### 3.2. Metabolomic analysis

**Figure 1:**
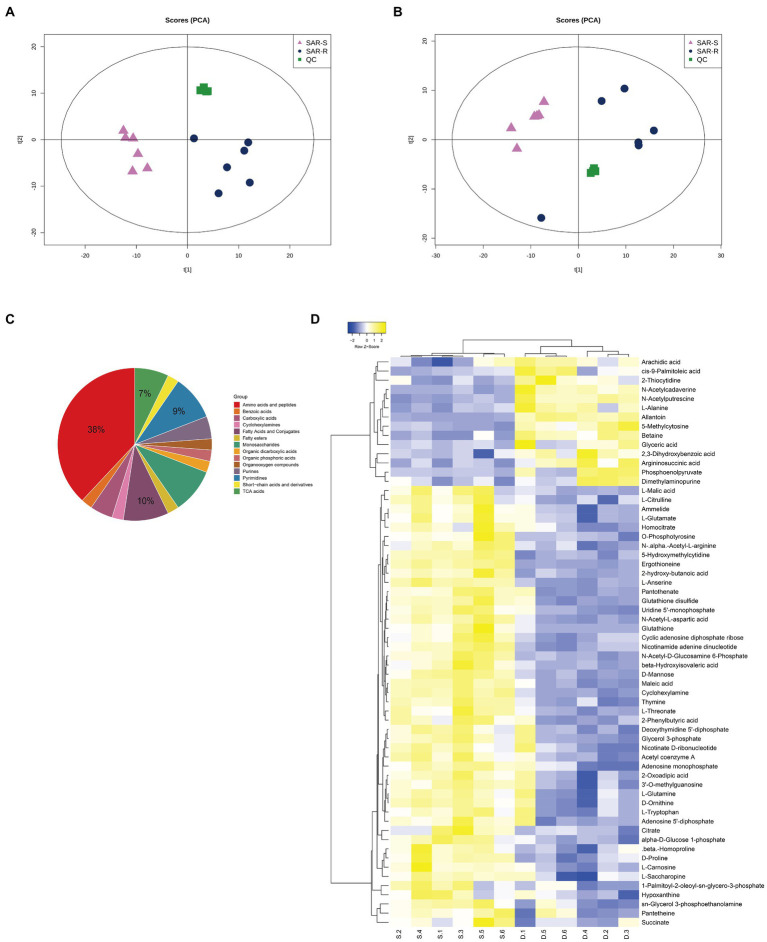
Analysis of metabolic profile. **(A)** PCA score of SAR-R and SAR-S in positive mode. **(B)** PCA score of SAR-R and SAR-S in negative mode. **(C)** Classification pie chart of all differential metabolites. **(D)** Cluster analysis of different metabolites. “D” represents sarafloxacin -induced resistant *Salmonella*, “S” represents sarafloxacin -sensitive *Salmonella*.

We used UHPLC-Q-TOF MS to perform non-targeted metabolomic analysis of SAR-S, SAR-R, and 6 biological repeats in each group to observe the metabolic changes caused by drug resistance. A total of 453 metabolites were identified, including carbohydrates, amino acids, lipids, nucleotides, etc. (Supplementary Table 2). In order to show the metabolic differences more clearly, Principal component analysis (PCA) was used to score the two strains. The results showed that the repeated experimental samples in each group had good aggregation, and the two groups were separated by the main component, indicating that the metabolic components of the two sample groups were significantly different. QC samples were also closely clustered (Figures 1A,B). To obtain more reliable and significantly different metabolites, we adopted OPLS-DA analysis, with VIP score > 1 and *p* value <0.05 as the screening criteria for significantly different metabolites. A total of 62 metabolites were selected for subsequent bioinformatics analysis (Supplementary Table 3), where the first four metabolites with the highest content were amino acids and peptides (38%), fatty acids and conjugates (10%), pyrimidines (9%), and TCA acids (7%; Figure 1C). To demonstrate the different metabolites more clearly, the hierarchical clustering of differential metabolites is plotted (Figure 1D). When we further analyzed these differential metabolites, it was found that there were some metabolites with reduced abundance, such as acetyl coenzyme A, succinate, citrate, L-glutamate, nicotinamide adenine dinucleotide, adenosine 5 ‘- diphosphate, hypoxantine, adenosine monophosphate, thymine, uridine 5’ - monophosphate, which belong to the following metabolic pathways: TCA cycle, glycolysis/gluconogenesis, alanine, aspartate and glutamate metabolism, oxidative phosphorylation, purine/pyrimidine metabolism. Therefore, we speculate that these metabolic pathways are affected during the evolution of cell resistance and should be responsible for the development of drug resistance. Among these, the TCA cycle is crucial to the biochemical metabolic system as the central link of bacterial metabolism. It can receive pyruvate produced by the glycolysis pathway to promote the absorption and metabolism of carbohydrates and the production of ATP. Furthermore, the intermediate products NADH and FADH are also important components of oxidative physiology and provide raw materials for the synthesis of alanine, aspartate, and glutamate (pyruvate, oxaloacetate, and 2-oxoglutarate) to ensure the raw materials (amino acid) for protein synthesis are sufficient. Aspartate and glutamine provide synthetic raw materials for nucleotides, and oxidative phosphorylation provides energy; however, these are all based on the normal operation of the TCA cycle. Therefore, we speculate that the weakening of the TCA cycle affects other biochemical metabolic processes, which delay the global metabolism of drug-resistant bacteria. The bacteria enter hibernation, thus resisting the pressure of antibiotics and producing a drug-resistant phenotype.

### 3.3. L-leucine specifically increased the susceptibility of drug-resistant *Salmonella* to sarafloxacin

**Figure 2:**
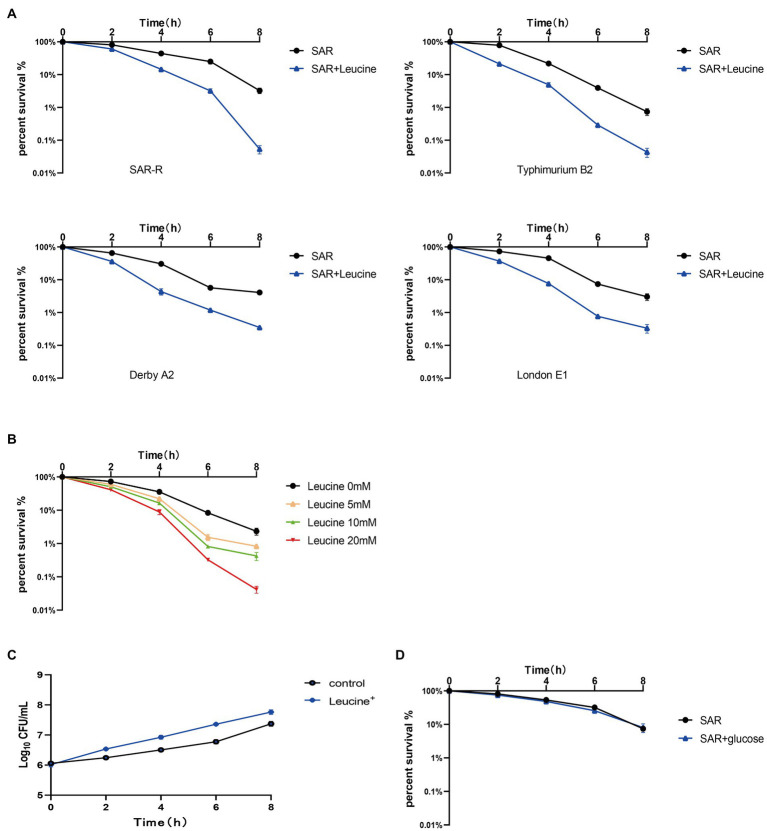
Killing curve of SAR-R and clinical drug-resistant *Salmonella*. **(A)** Percent survival curve of sarafloxacin (1MIC) and L-leucine (20 mM) action on four drug-resistant bacteria in M9 Minimal medium. **(B)** Percent survival curve of different concentrations of L-leucine (0, 5, 10, 20 mM) combined with sarafloxacin (1MIC) on SAR-R in M9 Minimal medium. **(C)** Growth promotion curve of glucose (40 mM) on SAR-R in M9 Minimal medium. **(D)** Percent survival curve of sarafloxacin (1MIC) and glucose (40 mM) action on SAR-R in M9 Minimal medium. Results are displayed as the mean ± SEM and three biological repeats are carried out.

The metabolic state of bacteria is crucial to the killing effect of antibiotics (Stokes et al., 2019). Many studies have shown that exogenous amino acids such as alanine, citrulline, and serine can modify the inhibited metabolic state, thereby promoting the killing effect of antibiotics on drug-resistant bacteria (Duan et al., 2016; Ye et al., 2018; Yong et al., 2021). Therefore, we speculate that a certain amino acid will modify the metabolic state of drug-resistant *Salmonella*, thereby synergizing the bactericidal effect of sarafloxacin. Then, we conducted a screening test on 20 protein amino acids. Among them, L-leucine proved to have the efficacy of synergizing with sarafloxacin in killing drug-resistant *Salmonella*. Furthermore, SAR-R, Typhimurium B2, Derby A2, and London E1 were incubated with sarafloxacin in M9 media with or without L-leucine for 8 h. The results showed that from the killing curve at each time point, the percent survival rate of the combination group (L-leucine plus sarafloxacin) was significantly lower than that of the sarafloxacin monotherapy group (Figure 2A). However, each strain’s most significant synergy time differed, which may be due to the different absorption and utilization efficiency of L-leucine. At a similar antibiotic concentration (1MIC), L-leucine with different concentrations (0, 5, 10, and 20 mM) showed dose-dependent manner synergistic effects (Figure 2B). Interestingly, when we increased the dose of L-leucine to 40 mM, the potential of L-leucine on sarafloxacin did not increase further, which may be due to the limited utilization of L-leucine by bacteria. Nevertheless, L-leucine, as an amino acid, can provide nutrients for the growth of bacteria, which may promote growth in a nutrient-poor medium and indirectly synergize the killing effect of bactericidal antibiotics. Therefore, to eliminate the role of L-leucine as a nutrient, we used glucose (the representative of nutrients) in combination with sarafloxacin to observe whether the same synergistic effect exists. The results indicate that the addition of glucose promoted the growth of bacteria but did not promote the bactericidal effect of sarafloxacin (Figures 2C,D). These results show that L-leucine specifically promoted the killing effect of sarafloxacin in drug-resistant *Salmonella*.

### 3.4. L-leucine increased intracellular NADH concentration

**Figure 3:**
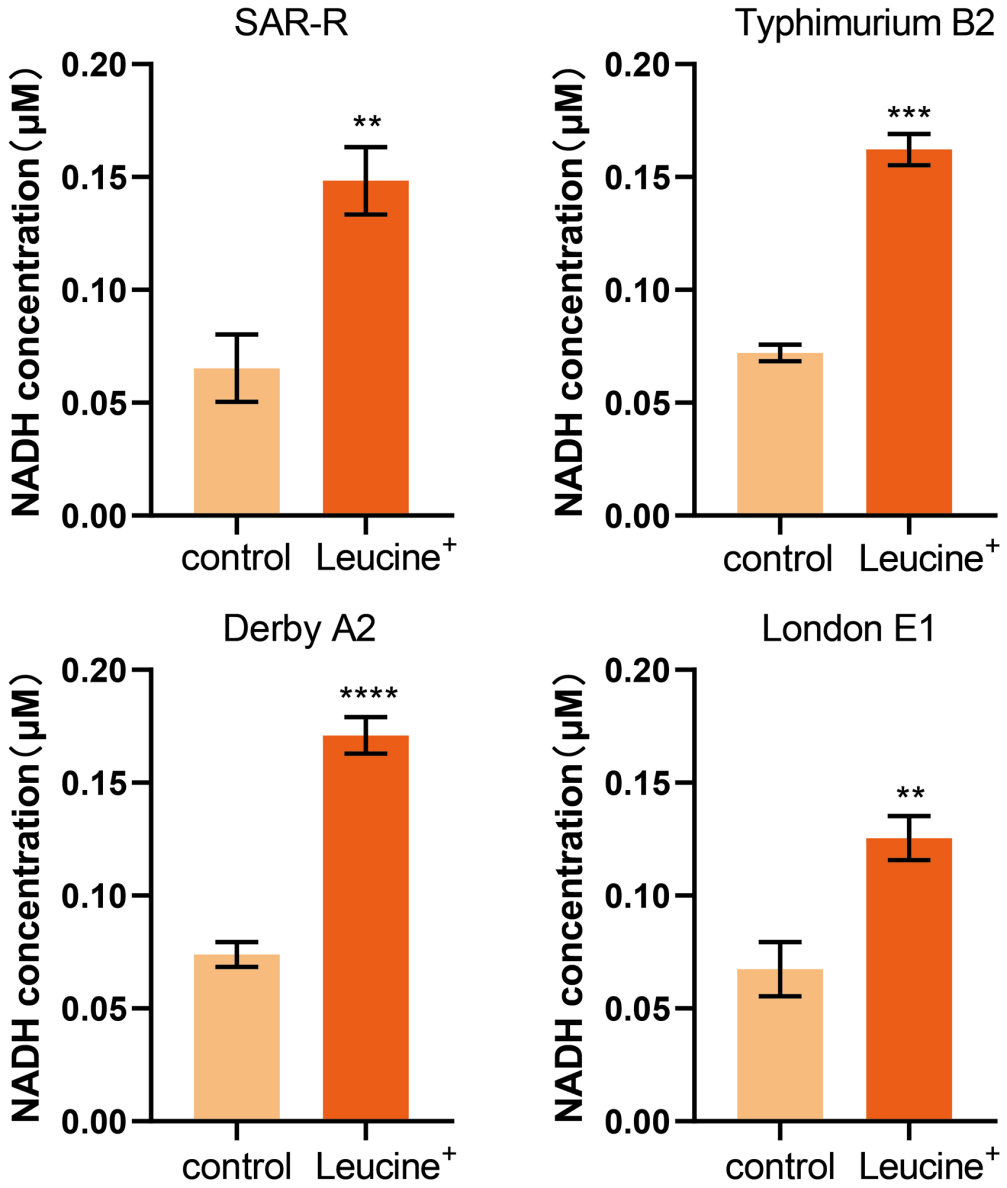
L-leucine increases the intracellular NADH concentration of SAR-R and clinical drug-resistant *Salmonella* in the presence 20 mM L-leucine. Results are displayed as the mean ± SEM and three biological repeats are carried out. Significant differences are identified (**p < 0.01), determined by unpaired *t*-test.

We speculated that exogenous L-leucine affects the TCA cycle and can stimulate drug-resistant bacteria since all carbon and nitrogen sources are ultimately metabolized in the TCA cycle. As a key intermediate, the increase in NADH concentration can indicate, to some extent, the activation of the TCA cycle. Therefore, we added L-leucine into the culture medium and measured the intracellular NADH concentration of bacteria after 4 h of cultivation. As shown in Figure 3, the NADH concentration of all drug-resistant bacteria increased after adding L-leucine compared with the control group. This indicates that L-leucine entered the TCA cycle and increased the NADH level.

### 3.5. L-leucine enhanced intracellular ATP concentration and activated the oxidative phosphorylation pathway

**Figure 4:**
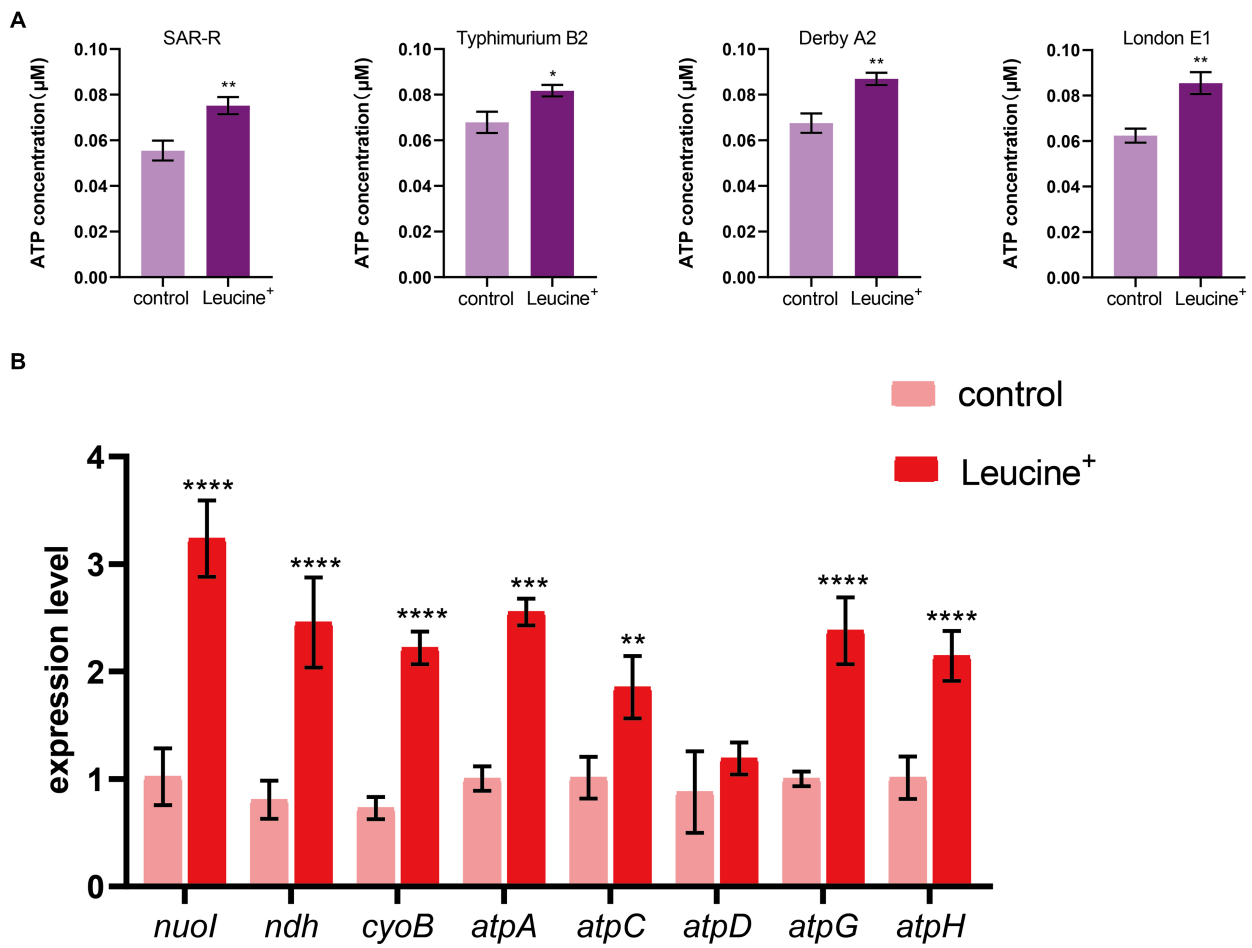
L-leucine activates electron transfer chain. **(A)** L-leucine increases the intracellular ATP concentration of SAR-R and clinical drug-resistant *Salmonella* in the presence 20 mM L-leucine. **(B)** RT-qPCR of key gene expression of electron transfer chain in SAR-R in the presence of 20 mM L-leucine. *nuoI*, NADH dehydrogenase I chain I; *ndh*, NADH:quinone reductase; *cyoB*, cytochrome o ubiquinol oxidase subunit I; *atpA*, *atpD*, *atpG*, *atpH* and *atpC*, membrane-bound ATP synthase, F1 sector, alpha-subunit, beta-subunit, gamma-subunit, delta-subunit, and epsilon-subunit. Results are displayed as the mean ± SEM and three biological repeats are carried out. Significant differences are identified (*p < 0.05, **p < 0.01, ***p < 0.001, ****p < 0.0001), determined by unpaired *t*-test.

The electron transport chain is an important pathway for cells to generate energy. When the electron transfer chain pumps electrons across the membrane to form a potential difference, ATP is synthesized by ATP synthase to fuel life activities. As the substrate of the electron transfer chain, NADH is also affected by L-leucine, which may increase the intracellular ATP concentration. Therefore, we added L-leucine into the culture medium and measured the intracellular ATP concentration at 4 h. As shown in Figure 4A, the intracellular ATP concentration increased significantly after adding L-leucine compared with the control group. This shows that the increased abundance of NADH entered the electron transfer chain and produced ATP, which improved the metabolic activity of the drug-resistant bacteria. To further verify the influence of the electron transfer chain, RT-qPCR was used to determine the expression level of key genes. As shown in Figure 4B, the expression of key genes *nuoI*, *ndh*, *cyoB*, *atpC*, *atpD*, *atpG*, and *atpH* all increased after L-leucine addition. These results showed that after L-leucine treatment, the oxidative phosphorylation pathway was activated, and the intracellular ATP concentration increased, indicating that the energy metabolism of drug-resistant bacteria was reprogrammed.

### 3.6. L-leucine stimulates the TCA cycle

**Figure 5:**
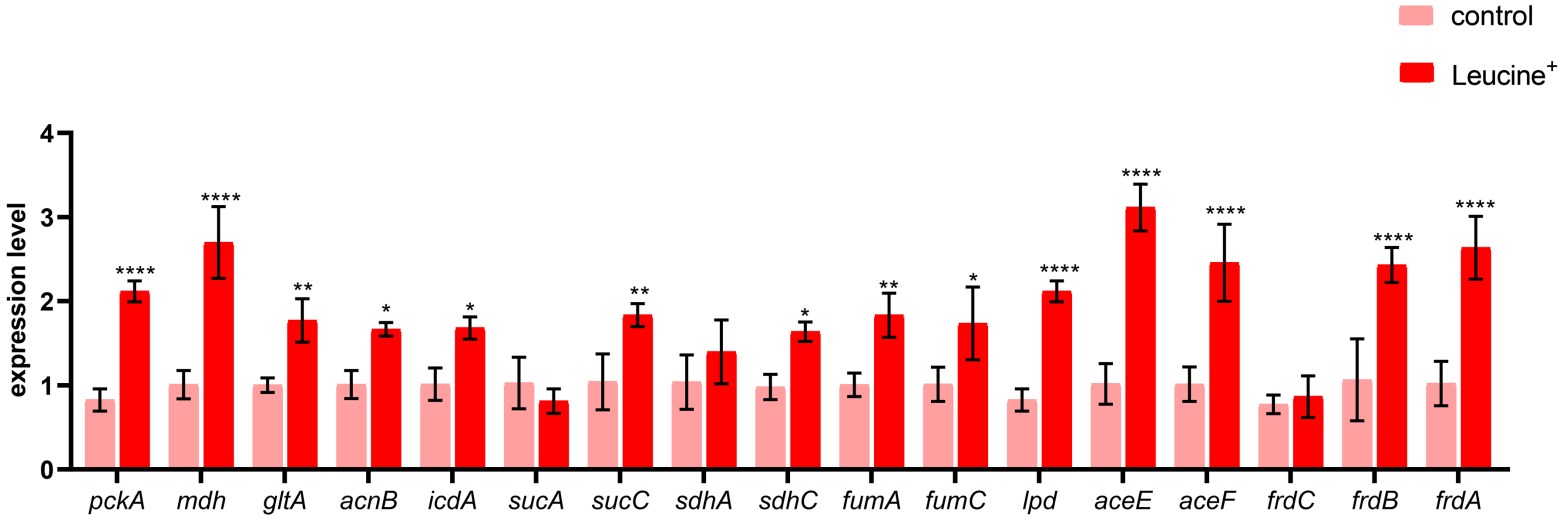
L-leucine activates TCA cycle. RT-qPCR of key gene expression of TCA cycle in SAR-R in the presence of 20 mM L-leucine. *pckA*, phosphoenolpyruvate carboxykinase; *mdh*, malate dehydrogenase; *gltA*, citrate synthase; *acnB*, aconitate hydratase 2; *icdA*, isocitrate dehydrogenase; *sucA*, 2-oxoglutarate dehydrogenase E1 component; *sucC*, succinyl-CoA synthetase beta subunit; *sdhA,* succinate dehydrogenase; *sdhC*, succinate dehydrogenase; *fumA*, fumarate hydratase, class I; *fumC*, fumarate hydratase, class II; lpd, dihydrolipoyl dehydrogenase; *aceE*, pyruvate dehydrogenase E1 component; *aceF*, pyruvate dehydrogenase E2 component; *frdC*, fumarate reductase subunitC; *frdB*, fumarate reductase iron–sulfur subunit; *frdA,* fumarate reductase flavoprotein subunit. Results are displayed as the mean ± SEM and three biological repeats are carried out. Significant differences are identified (*p < 0.05,**p < 0.01, ***p < 0.001,****p < 0.0001).

To confirm that the increased NADH and ATP levels were related to the TCA cycle, we measured the gene expression of key enzymes in the TCA cycle, pyruvate dehydrogenase (*aceE*, *aceF*), citrate synthase (*gltA*), isocitrate dehydrogenase (*icdA*), 2-oxoglutarate dehydrogenase E1 component (*sucA*), succinyl-CoA synthetase (*sucC*), dihydrolipoyl dehydrogenase (*lpdA*), succinate dehydrogenase (*sdhA*, *sdhC*), fumarate reductase (*frdA*, *frdB*, *frdC*), fumarate hydratase (*fumA*, *fumC*), and malate dehydrogenase (*mdh*). The expression level of these 15 genes is shown in Figure 5. After co-culturing bacteria with L-leucine for 6 h, the expression level of most genes increased significantly, suggesting that exogenous L-leucine promoted the function of the TCA cycle and that the increased NADH and ATP concentration was attributed to the activation of this cycle.

### 3.7. L-leucine increase the ROS level upon treatment with sarafloxacin

**Figure 6:**
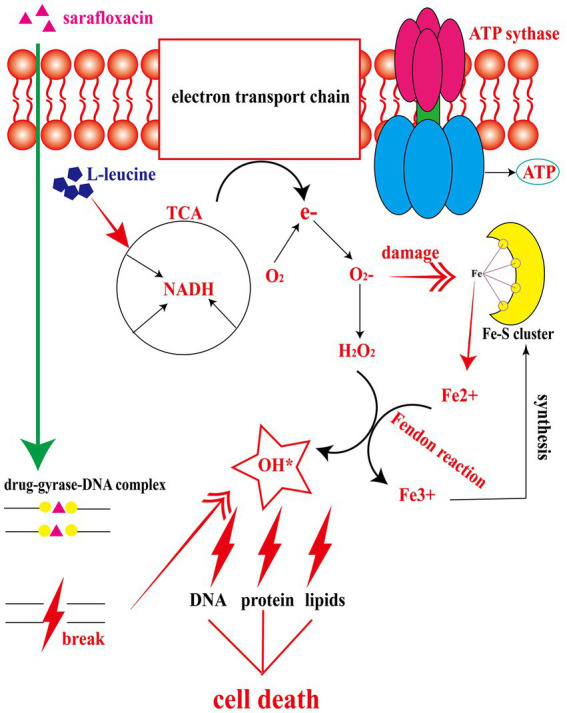
The mechanism of L-leucine promoting the killing effect of sarafloxacin on drug-resistant *Salmonella*. After L-leucine enters the cell, it stimulates TCA cycle and increases NADH content. NADH enters the electron transfer chain to accelerate electron transfer and ATP synthesis. The excessively activated electron transfer chain leads to electron leakage, and O_2_ receives e- to become superoxide, which then generates hydrogen peroxide under the catalysis of superoxide dismutases. Meanwhile, superoxide dismutases can disrupt the Fe-S cluster and produce free Fe^2+^. Fe^2+^and hydrogen peroxide produce a Fenton reaction, generating deadly hydroxyl radical to kill cells, while the oxidation product Fe^3+^continues to synthesize Fe-S cluster.

**Figure 7:**
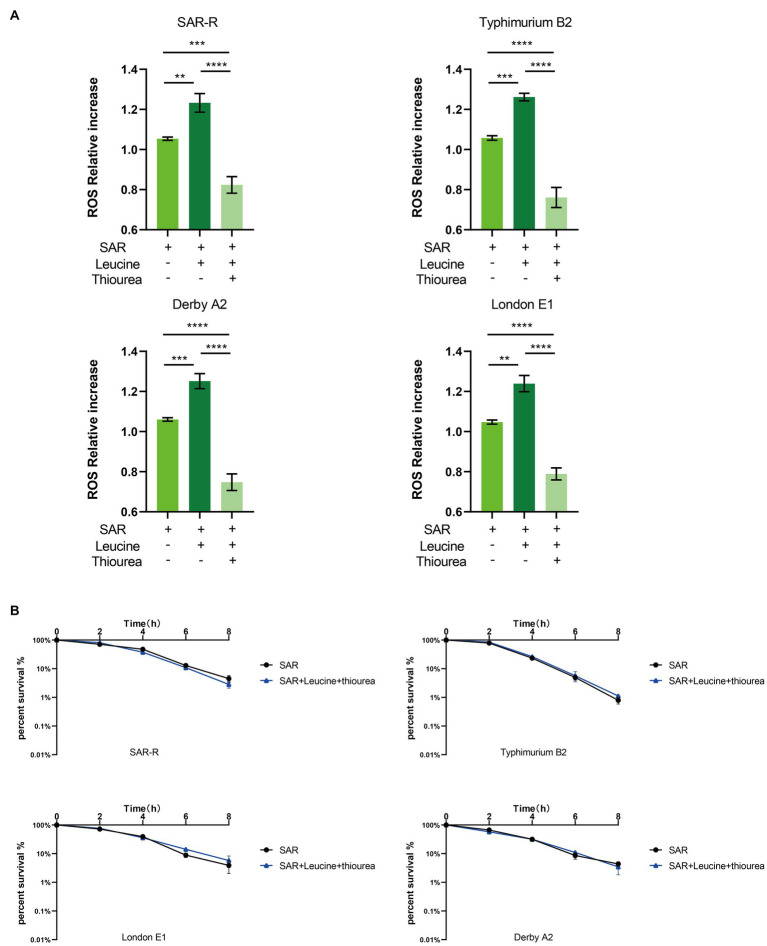
Intracellular ROS level of SAR-R and clinical drug-resistant *Salmonella*. **(A)** Combination of L-leucine and sarafloxacin enhanced ROS production. **(B)** Percent survival curve of 1 MIC and 20 mM L-leucine and 60 mM thiourea action on SAR-R and clinical drug-resistant *Salmonella* in M9 Minimal medium. Results are displayed as the mean ± SEM and three biological repeats are carried out. Significant differences are identified (**p < 0.01, ****p < 0.0001), determined by unpaired *t*-test.

Three types of bactericidal antibiotics, β-lactamides, aminoglycosides, and fluoroquinolones, promote the production of harmful hydroxyl radicals in bacterial cells destroying DNA, lipids, and proteins in cells, thus causing cell death (Kohanski et al., 2007). Quinolones combine with DNA gyrase and DNA topoisomerase IV to form a ternary complex during DNA replication. When DNA is degraded, superoxide is first generated, then peroxide through a disproportionation reaction, and finally, harmful hydroxy radicals through the Fenton reaction. Some studies have shown that electrons leak during the transmission of the respiratory chain and form superoxide (Kohanski et al., 2007). In this study, the activation of NADH and the electron transfer chain was confirmed. In order to explore whether L-leucine enhanced the killing effect of sarafloxacin by increasing endogenous ROS, we used a DCFH-DA fluorescence probe to detect ROS levels with or without L-leucine (Figure 6A). Compared with the sarafloxacin group alone, the ROS levels significantly increased. Consistently, when thiourea, a ROS eliminator, was added to the combined treatment group, the content of ROS content decreased significantly (Figure 7A). the enhanced bactericidal effect of L-leucine disappeared (Figure 7B). These results suggest that ROS plays an important role in the process of collaborative killing.

## 4. Discussion

Due to the overuse of clinical antibiotics, the prevalence of multidrug-resistant bacteria poses a huge threat to public health safety. Combining adjuvants and antibiotics may be safer and more effective than developing new antibiotics (Pieren and Tigges, 2012; Worthington and Melander, 2013). In this study, we first used half of the MIC concentration to obtained SAR-R and SAR-S. UHPLC-Q-TOF MS was used for the non-targeted metabolomic detection of SAR-S and SAR-R. The results showed significant metabolic differences caused by drug resistance. A total of 62 different metabolites were identified in the positive and negative ion modes. Then we found that some differential metabolites with reduced abundance were involved in the central carbon metabolism related metabolic pathway led by TCA. However, the abundance of phosphoenolpyruvate involved in the glycolysis process showed an increase, which may be due to the disorder of central carbon metabolism leading to the accumulation of intermediate products, further inhibiting the normal operation of the TCA cycle.

Various of studies have demonstrate improved killing by antibiotics following the reprogramming of deranged metabolism. Multiple amino acids have been confirmed to potentially modify metabolisms, such as, serine, citrulline, and alanine (Duan et al., 2016; Ye et al., 2018; Yong et al., 2021). We also tested a variety of amino acids in our experiments, but these above were not effectively repeated, which may be due to differences in experimental drugs, different experimental strains. In this study, we have measured the combined bactericidal efficacy of all 20 protein amino acids with sarafloxacin, including the above amino acids that have been proven to have synergistic effects. However, in all experiments, only the synergistic effect of L-leucine was confirmed. This may be due to the different absorption and utilization effects of various amino acids by different bacteria. We speculate that different drugs act differently on various bacteria, although most bacteria adjust their metabolic situation to lower their metabolism to evade the killing effect of antibiotics. However these alterations are special and we can only summarize several features in common, such as the attenuation of the central carbon metabolism. Specific alterations were unique in each sample. It is also interesting to note that several studies have demonstrated that sugars (glucose, fructose, D-ribose) can function as an antibiotic adjuvant (Peng et al., 2015; Su et al., 2015; Zhou et al., 2022), However, in this experiment glucose only promoted bacterial growth, and deficiency did not play a role in promoting killing, supporting the view that “every metabolic change that develops as a result of drug resistance is unique.” In summary, L-leucine was specifically selected during our experiments and targeted in our study targeted in our study.

Furthermore, we found that L-leucine promoted the killing effect of sarafloxacin against SAR-R and other clinically resistant *Salmonella Typhimurium* B2, Derby A2, and London E1 with different serotypes. However, each bacterium’s most effective synergistic killing time differed, which may be due to the different biochemical characteristics of each strain and the difference in the absorption rate and utilization efficiency of L-leucine. In order to exclude the role of L-leucine as a nutrient, we also used glucose as a control. The results were similar to those we predicted. Glucose did not show a synergistic effect, which also signifies the special role of L-leucine. We performed the same experiment on other fluoroquinolones (such as danooxacin). Interestingly, the results showed that only some strains had a synergistic killing effect, which may be attributed to the specific synergistic effect caused by different drug structures or the resistance of different strains to various fluoroquinolones, which needs further study.

In order to explore the specific mechanism of L-leucine in the cell, we measured the NADH and ATP concentrations and ROS levels of bacteria after cultivation with L-leucine. Overall, NADH, ATP, and ROS levels increased with L-leucine. To further confirm these results, we used RT-qPCR to determine the expression of each gene in the TCA cycle and electron transfer chain. The results showed that the expression level of most genes increased. Therefore, we concluded that exogenous L-leucine activated the TCA cycle after entering the cell membrane; thus, the abundance of NADH, the intermediate product of the TCA cycle, increased. When NADH entered the electron transfer chain as a substrate, it accelerated the electron transfer, increasing the ATP concentration from the product and correcting the inhibited carbon metabolism. Simultaneously, the function of the electron transfer chain accelerated, increasing the leakage of electrons, and the oxygen molecules received electrons and produced more ROS through the Fenton reaction, which promoted the death of bacteria (Figure 6). This was also confirmed by the ROS elimination agent, thiourea. When exogenous thiourea was added to the culture medium, the intracellular ROS level decreased rapidly, and the synergistic effect of L-leucine disappeared.

In addition to interacting with the target, bactericidal antibiotics will cause bacteria to produce ROS and destroy the cell structure (Kohanski et al., 2007). Many studies have also begun to look for antibiotic adjuvants at the metabolic level. Fructose, D-ribose, serine, alanine, thymine, citrulline, and glutamine as metabolites have been proven to reprogram the metabolic state of bacteria to improve the bactericidal effect of various antibiotics (Duan et al., 2016; Ye et al., 2018; Liu et al., 2021; Yong et al., 2021; Zhao et al., 2021; Zhou et al., 2022). However, most of these studies are aimed at aminoglycoside drugs, such as citrulline, glutamine and apramycin, D-ribose and gentamicin, alanine and kanamycin. This is due to the unique absorption mechanism of aminoglycoside drugs which is related to PMF. The generation of PMF is also associated with the electron transfer chain, as it is responsible for generating the potential difference in cell membranes. Ultimately, they are closely related to the TCA cycle because the substrate NADH of the electron transfer chain comes from the reaction catalyzed by isocitrate dehydrogenase and malate dehydrogenase in the TCA cycle. Fluoroquinolones are widely used in clinical antibacterial treatment, but the current drug resistance caused by overuse and misuse also encourages us to speed up the search for antibiotic adjuvants. Animals, especially edible livestock and poultry, are closely related to humans, and even some drug-resistant plasmids can be transmitted to humans to cause health and safety crises (Katale et al., 2020). As a special fluoroquinolone drug for animals, sarafloxacin is significant in finding an antibiotic adjuvant to relieve the pressure of clinical drug resistance, whether for aquaculture or public health safety.

In summary, non-targeted metabolomics was performed to determine the differential metabolites caused by drug resistance, and we further speculate that central carbon metabolism plays a crucial role in the evolution of drug resistance. It was also proved that L-leucine could increase the intracellular NADH and ATP concentration and ROS level of bacteria. This makes L-leucine specific in promoting the killing effect of sarafloxacin against drug-resistant *Salmonella*. Our results provide a theoretical basis for alleviating clinical drug resistance. It also provides evidence that L-leucine can be used as a potential adjuvant to enhance the efficacy of fluoroquinolones.

## Data availability statement

The datasets presented in this study can be found in online repositories. The names of the repository/repositories and accession number(s) can be found at: https://www.ebi.ac.uk/metabolights/; MTBLS7711, MTBLS7713.

## Author contributions

HY carried out the main experiments and data analysis and wrote the manuscript. YZ participated in the *in vitro* validation tests. QL and CZ participated in the isolation and identification of clinical drug-resistant bacteria. BF conceived and designed the experiments. All authors contributed to the article and approved the submitted version.

## Funding

This work was funded by the Local Innovative and Research Teams Project of Guangdong Pearl River Talents Program (No. 2019BT02N054).

## Conflict of interest

The authors declare that the research was conducted in the absence of any commercial or financial relationships that could be construed as a potential conflict of interest.

## Publisher’s note

All claims expressed in this article are solely those of the authors and do not necessarily represent those of their affiliated organizations, or those of the publisher, the editors and the reviewers. Any product that may be evaluated in this article, or claim that may be made by its manufacturer, is not guaranteed or endorsed by the publisher.
